# Simultaneous infection of Ixodes ricinus nymphs by two Borrelia burgdorferi sensu lato species: possible implications for clinical manifestations.

**DOI:** 10.3201/eid0103.950304

**Published:** 1995

**Authors:** B Pichon, E Godfroid, B Hoyois, A Bollen, F Rodhain, C Pérez-Eid

**Affiliations:** Unité d'Ecologie des Systèmes Vectoriels, Institut Pasteur, Paris, France.


### Simultaneous Infection of *Ixodes ricinus* Nymphs by Two *Borrelia burgdorferi* Sensu Lato Species: Possible Implications for Clinical Manifestations

Data from European studies indicate that in humans, particular *Borrelia burgdorferi* genospecies may be associated with specific clinical manifestations of Lyme disease. Infections by *B. burgdorferi* sensu stricto tend to lead to arthritic symptoms, whereas infections by *B. garinii* appear to cause neurologic complications. Late cutaneous manifestations (acrodermatitis) appear to be associated with *B. afzelii* (1). Mixed clinical manifestations have also been described (2). Recently it has been demonstrated, by using polymerase chain reaction (PCR), that DNA from more than one of the three *Borrelia* species associated with Lyme disease in Europe was present in the biological fluids of Lyme disease patients (3). These data raise questions concerning the relative growth of the *Borrelia* species after a bite by a dually infected tick, the clinical significance of human infection caused by more than one species of *Borrelia*, and the origin of these multiple infections. This last point evokes the following question: do they result from successive bites by two infected ticks or from a single bite by a tick infected by more than one species?

To investigate whether ticks are infected by different species of the *B. burgdorferi* complex at the same time, we carried out a survey of the vector *Ixodes ricinus* during the spring of 1994, in Rambouillet Forest near Paris. A total of 249 unfed nymphs, collected from vegetation, were analyzed by PCR. The ticks were then crushed in phosphate-buffered saline, solubilized in 0.5% Tween 20, and boiled for 10 min. The resulting lysate was used as a template for the amplification reactions by either the universal ospA-based primers SL or the three pairs of genospecific-based primers (3). These last primers distinguish the three Lyme disease-associated *B. burgdorferi* sensu lato species, i.e., *B. burgdorferi* sensu stricto, *B. garinii*, and *B. afzelii*. In some cases, amplified DNA products were digested with specific restriction enzymes to confirm the typing of the *Borrelia* strain.

Thirty of the 249 nymphs were positive for *B. burgdorferi* when SL universal primers were used. Further testing of 5 of 30 nymphs by PCR, using genospecific primer sets and restriction analysis, did not confirm the preliminary results with the universal primers. This may have been due either to the genotypic variability of *B. burgdorferi* sensu lato or to the existence of other distinct subgroups or genomic species included in *B. burgdorferi* sensu lato, as other data appear to indicate (4). Of the 25 other nymphs, 22 were analyzed by both restriction analyses and the specific primers, and three by restriction analysis alone. (The available tick material was not sufficient to perform PCR with genospecific primers.) Nineteen nymphs were infected by a single species of *Borrelia* (four by *B. garinii*, 15 by *B. afzelii*), and six were infected by more than one (two by both *B. burgdorferi* sensu stricto and *B. garinii*, three by *B. garinii* and *B. afzelii*, one by *B. burgdorferi* sensu stricto and *B. afzelii*).

From these results, it appears that when nymphs are infected with one species, *B. afzelii* is the most prevalent. This species may actually be prevalent in this study area or may have a greater tropism for dermal tissue and/or for the peripheral circulatory system of the vertebrate than the other two species. In infected nymphs, the simultaneous presence of more than one genospecies in unfed nymphs of *I. ricinus* was not exceptional (24%), and all combinations of two species were observed. The association of three genospecies has not yet been detected. Simultaneous infections in unfed nymphs could have different explanations. The first is a larval meal on a host infected by more than one species. Recently, *Apodemus speciosus* (field mice) infected by two different species have been found (5). A second possibility is successive infectious interrupted larval meals. A third possibility is an infectious larval meal by a previously transovarially infected larva. The fourth possibility is a mixed infection acquired transovarially.

**Bruno Pichon,* Edmond Godfroid,† Bernard Hoyois,† Alex Bollen,† Francois Rodhain,* and Claudine Pérez-Eid***

* Unité d'Ecologie des Systèmes Vectoriels, Institut Pasteur 75724 Paris, France

† Laboratoire de Genetique Appliqué, Université Libre de Bruxelles, Rue de l'Industrie 24, B-1400 Nivelles, Belgium

This work was supported by grants from the Recherche & Partage Association, the Gould Foundation, and the Conseil du Departement du Val d'Oise. E. Godfroid, B. Hoyois, and A. Bollen, were supported by a grant from the Walloon Region of Belgium (Convention UIB, Region Wallonne No. 2267).

### References

1. Assous MV, Postic D, Paul G, Nevot P, Baranton G. Western blot analysis of sera from Lyme borreliosis patients according to the genomic species of the *Borrelia* strain used as antigens. Eur J Clin Infect Dis 1993;12:261-8.

2. Wienecke R, Neubert U, Volkenandt M. Cross-immunity among types of *Borrelia burgdorferi*. Lancet 1993;341:830-1.
3. Demaerschalck I, Ben Messaoud A, De Kesel M, Hoyois B, Lobet Y, Hoet P, et al. Simultaneous presence of different *Borrelia burgdorferi* genospecies in biological fluids of Lyme disease patients. J Clin Microbiol 1995;33:602-8.
4. Nohlmans LMKE, De Boer R, Van Den Boggard AEJM, Van Boven CPA. Genotypic and phenotypic analysis of *Borrelia burgdorferi* isolates from the Netherlands. J Clin Microbiol 1995;33:119-25.
5. Nakao M, Miyamoto K. Mixed infection of different *Borrelia* species among *Apodemus speciosus* mice in Hokkaido, Japan. J Clin Microbiol 1995;33:490-2.

## References

[PDF_00105] Assous M. V., Postic D., Paul G., Névot P., Baranton G. (1993). Western blot analysis of sera from Lyme borreliosis patients according to the genomic species of the Borrelia strains used as antigens.. Eur J Clin Microbiol Infect Dis.

[PDF_00115] Demaerschalck I., Ben Messaoud A., De Kesel M., Hoyois B., Lobet Y., Hoet P., Bigaignon G., Bollen A., Godfroid E. (1995). Simultaneous presence of different Borrelia burgdorferi genospecies in biological fluids of Lyme disease patients.. J Clin Microbiol.

[PDF_00124] Nakao M., Miyamoto K. (1995). Mixed infection of different Borrelia species among Apodemus speciosus mice in Hokkaido, Japan.. J Clin Microbiol.

[PDF_00120] Nohlmans L. M., de Boer R., van den Bogaard A. E., van Boven C. P. (1995). Genotypic and phenotypic analysis of Borrelia burgdorferi isolates from The Netherlands.. J Clin Microbiol.

